# A Few Stickleback Suffice for the Transport of Alleles to New Lakes

**DOI:** 10.1534/g3.119.400564

**Published:** 2019-12-04

**Authors:** Jared Galloway, William A. Cresko, Peter Ralph

**Affiliations:** * Institute of Ecology and Evolution and; † Department of Mathematics, University of Oregon

**Keywords:** local adaptation, threespine stickleback, genomic simulation

## Abstract

Threespine stickleback populations provide a striking example of local adaptation to divergent habitats in populations that are connected by recurrent gene flow. These small fish occur in marine and freshwater habitats throughout the Northern Hemisphere, and in numerous cases the smaller freshwater populations have been established “de novo” from marine colonists. Independently evolved freshwater populations exhibit similar phenotypes that have been shown to derive largely from the same standing genetic variants. Geographic isolation prevents direct migration between the freshwater populations, strongly suggesting that these shared locally adaptive alleles are transported through the marine population. However it is still largely unknown how gene flow, recombination, and selection jointly impact the standing variation that might fuel this adaptation. Here we use individual-based, spatially explicit simulations to determine the levels of gene flow that best match observed patterns of allele sharing among habitats in stickleback. We aim to better understand how gene flow and local adaptation in large metapopulations determine the speed of adaptation and re-use of standing genetic variation. In our simulations we find that repeated adaptation uses a shared set of alleles that are maintained at low frequency by migration-selection balance in oceanic populations. This process occurs over a realistic range of intermediate levels of gene flow that match previous empirical population genomic studies in stickleback. Examining these simulations more deeply reveals how lower levels of gene flow leads to slow, independent adaptation to different habitats, whereas higher levels of gene flow leads to significant mutation load – but an increased probability of successful population genomic scans for locally adapted alleles. Surprisingly, we find that the genealogical origins of most freshwater adapted alleles can be traced back to the original generation of marine individuals that colonized the lakes, as opposed to subsequent migrants. These simulations provide deeper context for existing studies of stickleback evolutionary genomics, and guidance for future empirical studies in this model. More broadly, our results support existing theory of local adaptation but extend it by more completely documenting the genealogical history of adaptive alleles in a metapopulation.

The canonical model for the genetics of adaptation has long been the sequential fixation of new mutations (Maynard Smith and Haigh 1974; Endler 1977; Orr 2005). While it has proved to be a useful baseline for understanding the genetic variation we see in species today, this model is now rightfully understood as incomplete for many species in nature that have more complicated population structures (Lai *et al.* 2019; Schrider and Kern 2017). In particular, empirical studies have increasingly identified the need to more deeply incorporate standing genetic variation into adaptation dynamics for metapopulations inhabiting an array of habitats (Hermisson and Pennings 2005; Barrett and Schluter 2008). Populations experiencing diverse selective pressures while still exhibiting significant gene flow often result in more complex genomic signals that are still not fully understood (Charlesworth 1997; Charlesworth *et al.* 2003; Nosil *et al.* 2009; Flaxman 2013; Samuk *et al.* 2017). Concurrently, a growing number of empirical studies have identified instances of convergent evolution using standing genetic variation (Jones *et al.* 2012; Roesti *et al.* 2012; Feulner *et al.* 2015; Schrider and Kern 2017; Barrett and Schluter 2008; Nelson and Cresko 2018; Nelson *et al.* 2019; Bassham *et al.* 2018). However, it is still not clear how variation in evolutionary processes – such as gene flow, recombination, selection and mutation – can promote the maintenance and re-use of standing genetic variation, particularly during colonization and adaptation to new environments (Nelson and Cresko 2018; Pritchard 2010; Yeaman and Whitlock 2011; Schrider and Kern 2017). It is similarly unclear whether variation in these evolutionary processes can determine the genetic architecture of evolving traits via standing genetic variation. Theoretical work is mostly limited to one or two loci, or relies on approximations of uncertain validity (*e.g.*, Slatkin 1975; Petry 1983; Barton and Bengtsson 1986; Yeaman and Whitlock 2011; Ralph and Coop 2015). Therefore, detailed simulation models of specific systems can provide an important complement to empirical studies in the lab and field, by helping us understand precisely how standing genetic variation might fuel local adaptation, and what genomic signals we can expect to see.

The ancestral marine form of threespine stickleback fish (*Gasterosteus aculeatus*) has given rise to hundreds of thousands to millions of independently derived freshwater populations in recently de-glaciated regions around the Northern Hemisphere (Bell and Foster 1994; Thompson 1997; Cresko *et al.* 2007; Hunt *et al.* 2008). This model organism has provided some of the earliest data showing the heterogeneous nature of divergence across genomes and the much more extensive use of standing genetic variation than once thought (Schluter and Conte 2009; DeFaveri *et al.* 2011, 2013; Roesti *et al.* 2014; Nelson and Cresko 2018; Bassham *et al.* 2018; Nelson *et al.* 2019; Hohenlohe *et al.* 2010; Terekhanova *et al.* 2014; Marques *et al.* 2016). While geographic isolation often prevents direct migration between freshwater populations, threespine stickleback in them frequently evolve similar phenotypes (Cresko *et al.* 2004; Colosimo *et al.* 2004; Stuart *et al.* 2017; Hanson *et al.* 2016, 2017; Hirase *et al.* 2014). The most recent evolutionary genomic studies on stickleback document that while the overall dynamic of local adaptation to marine and freshwater habitats has been occurring for millions of years (Nelson and Cresko 2018; Nelson *et al.* 2019), independent local adaptation of marine individuals to freshwater environments has been observed to take place in just tens of generations (Terekhanova *et al.* 2014; Lescak *et al.* 2015; Bassham *et al.* 2018). For example, in 1964 the Great Alaskan Earthquake caused an uplift of many islands and coastal regions throughout the Gulf of Alaska. Studies of stickleback populations on uplifted Middleton Island showed that newly created freshwater ponds were invaded by the surrounding marine population of stickleback which evolved the freshwater syndrome of phenotypes in less than 50 years (Lescak *et al.* 2015; Bassham *et al.* 2018). Amazingly, the portions of the genomes of these populations that showed increased divergence from the oceanic population mirrored those previously found to differ between ocean and freshwater populations that that have been geographically separated for thousands of years (Hohenlohe *et al.* 2010; Bassham *et al.* 2018).

But how can evolution occur at such a rapid pace? Waiting for new mutations to arise in each lake or pond would take much longer than the decades since the Alaskan earthquake with any plausible target size. Even more improbable is having divergence cluster in such similar genomic regions across independent populations. An alternative hypothesis is that the majority of alleles important for freshwater adaptation are present in the marine individuals due to recurrent gene flow from freshwater back in to marine populations. Schluter and Conte (2009) proposed a conceptual model they termed the “transporter” hypothesis to describe the process by which alleles beneficial in freshwater environments are maintained at migration-selection balance in the larger oceanic population and therefore available to be utilized during subsequent adaptation to new freshwater habitats. (The alleles conferring adaptation to freshwater environments are thereby “transported” to and reassembled in new lakes.) The first clear example of the global reuse of such alleles in stickleback was the gene *eda* which has been shown to be an important regulator for the number of bony lateral plates (Colosimo *et al.* 2004; Cresko *et al.* 2004; Colosimo *et al.* 2005). While the low lateral plate version of this gene arose millions of years ago, it is found in much younger freshwater ponds around the Northern Hemisphere (O’Brien *et al.* 2015). More recently, genome-wide haplotype analyses have provided evidence that *most* regions of the genome that distinguish marine-freshwater genetic differences share this pattern (Nelson and Cresko 2017).

While the growing body of population genomic data on stickleback evolution supports the transporter hypothesis, a number of questions remain. What are the actual population sizes, migration rates, and fitness differentials consistent with this hypothesis? How many differentially selected alleles exist, how many are used at any one time, and how are they arranged within the genome? A curious natural history observation underlying many of these questions is the fact that some newly formed freshwater habitats, such as the ponds on Middleton Island, are quite small and presumably the number of initial marine migrants is few. The variation carried by these few initial migrants might therefore be a small subset of the total variation, and thus be insufficient to fuel adaptation without subsequent influx of alleles from additional generations of marine migrants carrying the remaining freshwater adaptive alleles.

Here, we use individual-based forward simulations implemented in SLiM that incorporates selection on a quantitative trait explicitly determined additively from a realistically long genome to model the stickleback metapopulation and address these questions (Haller and Messer 2017, 2018). We ask how variation in amount of gene flow affects the genetic architecture of local adaptation to newly created freshwater ponds. Because we record the entire genealogy of all alleles (Kelleher *et al.* 2018), we can determine the distribution and abundance of haplotypes across all marine and freshwater populations to know the timing and proportion of potentially adaptive alleles that are actually utilized in each population. In addition, we can document how these adaptive alleles are distributed across the genomes, and as a consequence determine how this may affect the efficacy of genome scans for between-habitat differentiation.

## Methods

To explore these questions, we used SLiM (Haller and Messer 2017, 2018) to implement forward-time simulation of populations of individuals with explicitly represented genomes in which selection acted upon a single continuous quantitative trait. The details of the model were motivated by current understanding of threespine stickleback evolutionary, history and demography. The following model included divergent selection in the two habitats which had substantial spatial structure. Certain aspects of the model remain simplistic due to computational constraints; in particular, total population sizes are much smaller than in reality, although we may capture a good picture of local dynamics.

### Habitat and geography

**Figure 1:**
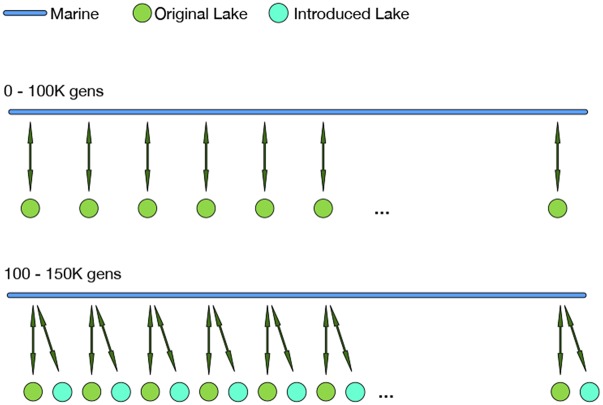
Diagram of simulated populations: a single, continuous, one-dimensional marine habitat (blue) is coupled to “lakes”, in which stickleback randomly mate, at discrete locations with arrows representing migration patterns. After an initial period of 100K generations with 25 lakes, an additional 25 lakes are added (at the same set of locations) and populated with marine individuals to simulate the appearance of newly accessible freshwater habitats colonized by marine stickleback. The marine habitat, and each set of 25 lakes, each contain 5,000 individuals at all times.

Our simulations include two habitat types – marine and freshwater – defined by the nature of their selective pressures, each with 5,000 diploid individuals. The arrangement of these habitats, depicted in Figure 1, roughly models a set of freshwater habitats along a stretch of coastline. The marine habitat is a continuous, one-dimensional range of length 25 units, while the freshwater habitat is divided into 25 discrete subpopulations (which we call “lakes”), each connected to the marine habitat at regularly spaced intervals (positions i−1/2 for 1≤i≤25).

Divergent selection is mediated by a single quantitative trait with different optima in marine and freshwater habitats. This situation roughly models the cumulative effect of the various phenotypes thought to be under divergent selection between the habitats, such as armor morphology, body size, craniofacial variation and opercle shape. The optimal trait values in the marine and freshwater habitats are +10 and −10 respectively, and fitness of a fish with trait value $x_{\text{ind}}$ in a habitat with optimal value $x_{\text{opt}}$ is determined by a Gaussian kernel with standard deviation 15, *i.e.*,

$$f\left(x_{\text{ind}};x_{\text{opt}}\right)=\text{exp}\left\{-\frac{1}{2}{\left(\frac{x_{\text{ind}}-x_{\text{opt}}}{15}\right)}^{2}\right\}.$$

Thus, the difference between an individual’s trait value and the optimum determines that individual’s fitness. We chose the difference between optima and strength of stabilizing selection in each habitat so that (a) around 10 (diploid, homozygous) mutations were sufficient to move from one optimum to the other, and (b) well-adapted fish from one habitat would have low, but nonzero, fitness in the other habitat.

### Genetic architecture of the trait

Individuals are diploid, with a linear chromosome $10^{8}$ bp long, with recombination events occurring at a rate of $10^{-8}$ per bp per generation. Mutations that can affect the trait under selection occur at rate $10^{-10}$ per locus per generation in ten regions of $10^{5}$ loci each, spread evenly along the chromosome. This translates to a total adaptive target size of $10^{-4}$ per individual per generation, or ≈1 trait-affecting mutation, per population of 5,000 diploids, per generation. This architecture was chosen to model a moderate number of clusters of trait-affecting genes, each of which having about 1Kb of potentially adaptive target (since mutation rates are closer to $10^{-8}$, a mutation rate of $10^{-10}$ is roughly equivalent to having potentially adaptive sites at one of every 100 bases). Each mutation in these regions is either additive, completely recessive, or completely dominant (with equal probability). Effect sizes for these mutations are chosen randomly from an exponential distribution with mean 1/2, either positive or negative with equal probability. Individual trait values ($x_{\text{ind}}$) are determined additively from the diploid genotypes. Concretely, an individual that is heterozygous and homozygous for mutations at sets of loci *H* and *D* respectively has trait value $x_{\text{ind}}=\sum_{i\in H}h_{i}s_{i}+\sum_{j\in D}s_{j}$, where $h_{i}$ and $s_{i}$ are the dominance coefficient and the effect size of the mutation at locus *i*. Subsequent mutations at the same locus replace the previous allele.

### Population dynamics

We use SLiM to simulate a Wright–Fisher population with non-overlapping generations and a fixed population size of 5,000 diploid individuals in each habitat. Each generation, the two parents of each new offspring are chosen proportional to their fitness (unlike actual stickleback, all individuals are hermaphroditic), and the contributing genomes are produced by Poisson recombination with an average of one crossover per chromosome per generation ($10^{-8}$ per locus per generation). Since the total population across *all* 25 lakes is fixed at 5000, and the Wright–Fisher model assumes unrealistic global population regulation, we normalize the fitnesses of each individual so that approximately 200 offspring are generated in each lake, each generation. (A simpler implementation would use SLiM’s “non-Wright-Fisher” model type, which was not available when we wrote the simulations; but we do not expect the difference to affect results.) To do this, we divide fitness values of each freshwater individual by the mean fitness in their lake, so that the mean fitnesses of all lakes are equal before selection happens.

As depicted in Figure 1 the marine population is continuous (one-dimensional) while lakes are discrete. Dispersal occurs both locally along the coastline within the marine habitat, as well as between the marine habitat and the lakes, with a lake–ocean migration rate denoted *m*. No migration occurs directly between the freshwater populations. All individual dispersal events can be thought of as occurring at the juvenile stage in the life cycle of the simulation. Each new individual in each habitat has parents from the other habitat with probability *m* (in which case we call it a “migrant”), and parents from the same habitat with probability 1−m. The first parent of each non-migrant individual in the freshwater habitat is chosen from the freshwater habitat proportional to fitness, and a mate is chosen from the same lake as the first, also proportional to fitness. The offspring then lives in the same lake as the parents. Parents for each non-migrant marine individual are chosen similarly: first, a single parent is chosen proportionally to fitness in the marine habitat, and then a mate is chosen, also proportionally to fitness but re-weighted by a Gaussian function of the distance separating the two, with standard deviation 1/2. Concretely, if the first parent is marine individual *i*, then marine individual *j* is chosen as the mate with probability proportional to $f\left(x_{j}\right)\text{exp}\left(-2d_{ij}^{2}\right)$, where $f\left(x_{j}\right)$ is the fitness of individual *j* and $d_{ij}$ is the distance between the two locations. Finally, each new marine offspring is given a position displaced from the first parent’s position by a random Gaussian distance with mean 0 and standard deviation 0.5, and reflected to stay within the habitat range. Parents for each freshwater migrant are chosen in the same way as for non-migrant marine individuals, and are assigned to the lake nearest to the position of the first marine parent. Similarly, parents for each marine migrant are both chosen from the same lake as before, and the offspring is given a spatial location in the marine habitat at the location of the parent’s lake.

### Colonization of newly formed lakes

To study how marine-derived populations adapt after colonizing newly appearing freshwater habitats, we introduce a new set of 25 lakes after 100,000 generations. These new lakes are populated with marine individuals to emulate a freshwater lake being colonized by oceanic stickleback that had previously been exchanging alleles with older freshwater populations. This creates two *sets* of lakes, which along with the marine population have a total of 15,000 individuals. Since this introduction of lakes doubles the number of lake-to-marine immigrants, the probability that a new marine individual has freshwater parents is 2m instead of *m*.

### Recording genealogical history

We used SLiM’s ability to record *tree sequences* (Haller *et al.* 2019; Kelleher *et al.* 2016) to output the genealogical history of all individuals at the time of introduction of new lakes, at the time of adaptation, and at the end of the simulation. This allowed us to directly query the true origins of adaptive alleles. In addition, it allowed for much larger simulations by avoiding the computationally expensive task of simulating neutral mutations which were retroactively added to the gene trees at a rate of $10^{-8}$ per locus per generation, as described in Kelleher *et al.* (2018). The tree sequence output by each simulation allows us to explore the origin of the genetic basis of adaptation in the new lakes. To do this, we constructed the genealogical tree relating all extant chromosomes at each locus along the genome. Using these trees we classified each adaptive allele, in each genome in the new lakes at the time of adaptation, into four categories:

1. *a “De novo” allele:* deriving from a new mutation that occurred in a new lake.
2. *a “Migrant” allele:* deriving from a migrant not in the initial generation that colonized the lake
3. *a “Captured” allele:* present in initial colonists of the new lake, and both common (above 50%) in the original lakes, and uncommon (below 50%) in the ocean.
4. *a “Marine” allele:* present in initial colonists of the new lake, but not a “captured” allele.

The proportion of trait-affecting alleles in new lakes that fall in these categories measures the degree to which selection in the new environments made use of (1) new mutation, (2) post-colonization migration, (3) standing variation at migration–selection balance, and (4) standing variation at mutation–selection balance.

We used neutral mutations to calculate $F_{ST}$ for each locus, which we then averaged in windows as a measure of between-population relative differentiation. Concretely, if $p_{f}$ and $p_{m}$ are the frequencies of a given mutant allele in the freshwater and marine habitats, respectively, and $\bar{p}=\left(p_{f}+p_{m}\right)/2$, then we compute $F_{ST}$ for that mutation as $1-\left(p_{f}\left(1-p_{f}\right)+p_{m}\left(1-p_{m}\right)\right)/\left(2\bar{p}\left(1-\bar{p}\right)\right)$ (Gillespie 1998).

### Data availability

The source code used to run and analyze the simulations in this paper are available at https://github.com/jgallowa07/SticklebackPaper. Supplemental material available at figshare: https://doi.org/10.25387/g3.10876052.

## Results & Discussion

To observe the impact of gene flow on selection in the new lakes, we varied the ocean–lake migration rate, *m*, across separate simulations from $5\times 10^{-5}$ to $5\times 10^{-1}$. Below, we often refer to these migration rates in terms of the number of migrants per lake, per generation, which we denote *M*. Since each lake contains 200 individuals, M=200m. Many aspects of adaptation changed substantially across this range, including the speed of adaptation, degree of sharing of adaptive alleles among lakes, and the population genetic signals left behind. At very low rates of gene flow, each new lake’s population adapted almost completely independently through *de novo* mutation, which took a very long time (≈20,000 generations). At very high rates of gene flow, local adaptation was constrained by the large influx of locally maladaptive alleles. Between these two extremes, genetic variation that allowed adaptation to freshwater habitats could move relatively easily between lakes. Perhaps surprisingly, only a few migrants per generation from the lakes to the ocean were needed to maintain sufficient genetic variation in the ocean to dramatically accelerate adaptation in new lakes.

### Rapid local adaptation at intermediate gene flow rates

**Figure 2:**
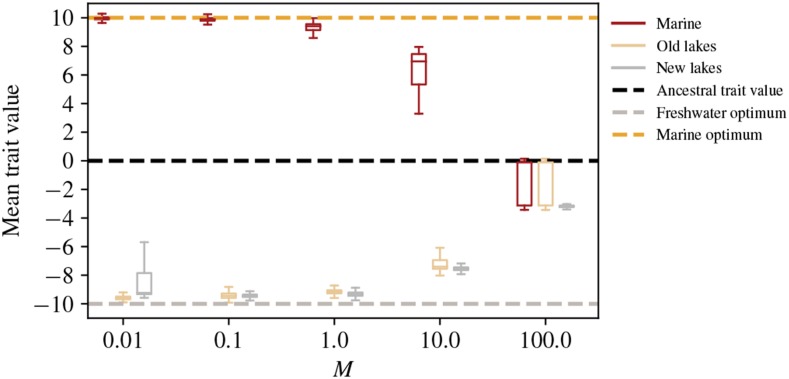
Distribution of mean individual trait values across generations of the simulation (recording started after introduction of the new lakes at 100,000 generations), for different migration rates. The dashed yellow and gray lines at ±10 give the optimum phenotypes in the marine and freshwater environments, respectively.

**Figure 3:**
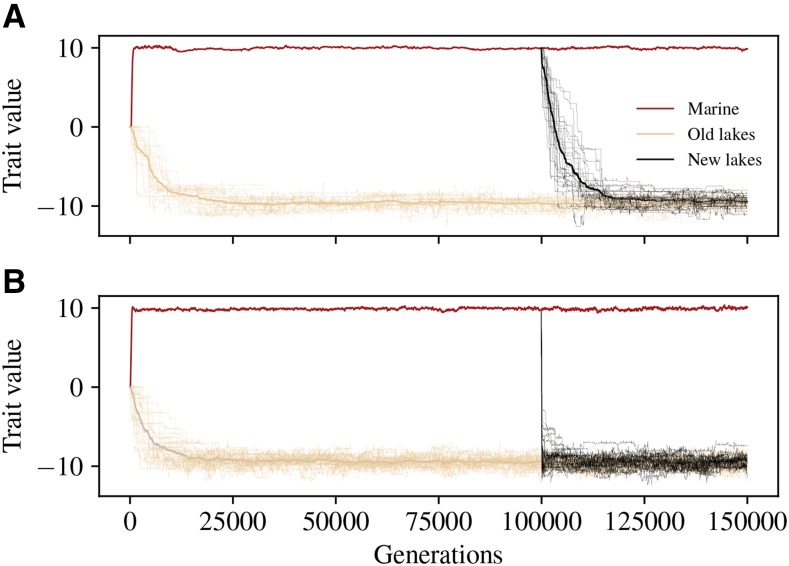
Mean individual trait values in the marine habitat (red line), the original lakes (tan lines; average thicker), and the new lakes (black lines; average thicker), across the course of two simulations, with migration rates of (A)M=0.01 and (B)M=0.1 migrants per lake per generation, respectively. Optimal trait values in the two habitats are at ±10. Analogous plots for other migration rates are shown in Figure S3.

**Figure 4:**
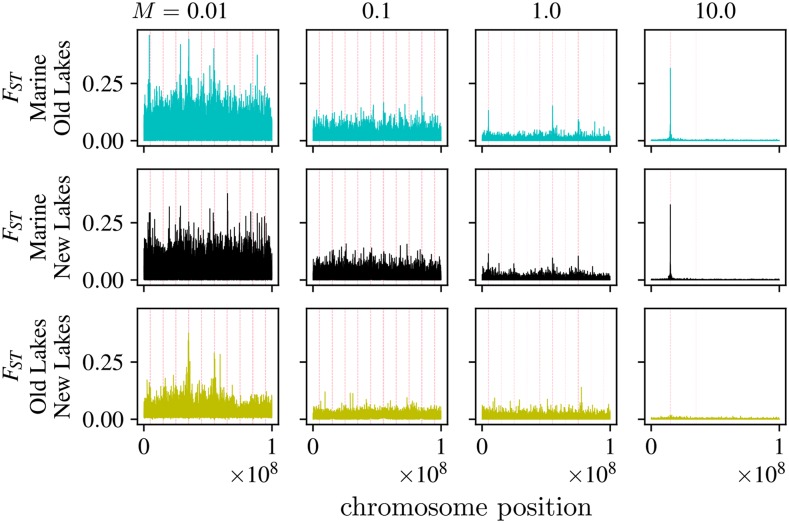
Average $F_{ST}$ in windows of 500bp between: (top) marine habitat and old lakes; (middle) marine habitat and new lakes; and (bottom) old and new lakes. The columns are ordered by *M* (the number of migrants per lake, per generation) and show $F_{ST}$ values for a separate simulation, $F_{ST}$ is calculated between all marine individuals, and all lakes pooled together. All locations of pre-existing freshwater adapted alleles have been highlighted by partially transparent pink dashed vertical lines, so darker shades of pink show more freshwater adapted alleles at that location.

Local adaptation occurred in all simulations with the exception of the highest gene flow at which half of each population was composed of migrants, as can be seen by the mean trait values of Figure 2. Figures 3 and S3 show how mean trait values in freshwater and marine populations diverged over time, until the trait means were close to the optimal values in each habitat. Establishment of adaptive alleles in the old lakes during the first few thousand generations manifests as jumps in the mean trait value – which move the trait by an amount of order 1 every few hundred generations (Figure 3). At the lowest rate of gene flow, M=0.01, differences at around 20 commonly polymorphic sites (about 10 that shift the trait in each direction) were responsible for most of the adaptive differences between freshwater and marine habitats. As expected, increasing migration rate decreased differentiation between habitats: as seen in Figure 4, $F_{ST}$ between marine and freshwater habitats at neutral sites steadily declines as migration increases.

**Figure 5:**
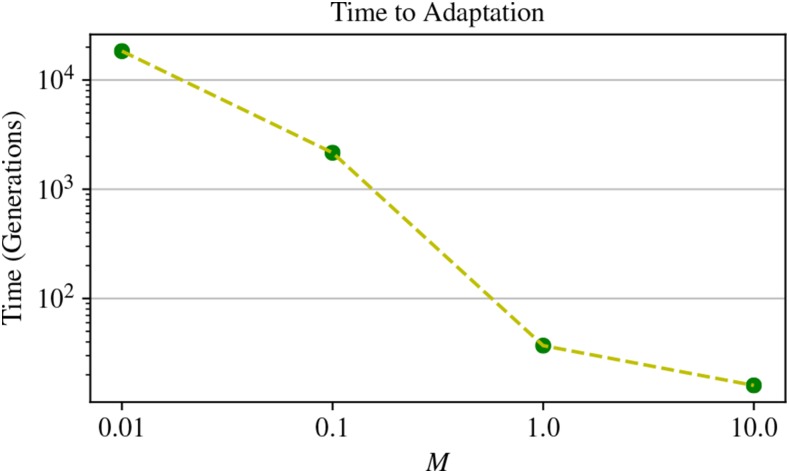
Time to adaptation as a function of migration rate. The time to adaptation is measured as the number of generations until the introduced population’s mean phenotype comes within 0.5 of the original lakes average phenotype. Each point represents a single simulation run. (Adaptation did not occur at the highest rate of gene flow.)

Adaptation occurred much more quickly at higher migration rates, both in the old and new sets of lakes. We measured this “time to adaptation” as the number of generations until average trait values in old and new lakes were within 0.5 of each other, shown in Figure 5 for different rates of gene flow. Adaptation of new lakes took over 18,000 generations at the lowest rate of M=0.01, while at M=1 migrant per lake per generation, new lakes adapted in just under 60 generations.

### Widespread allele sharing among lakes

Adaptive alleles were shared between lakes at many rates of gene flow, but not at the lowest. At low migration rates, the *initial* period of adaptation takes roughly 25 times longer for lakes than it does for the ocean. This difference occurs because in the absence of gene flow, each lake must wait for its own novel mutations to arise in order to adapt. Because the marine habitat is continuous, with 25 times more individuals than any one lake, there is a much larger influx of new mutations to be selected upon. At higher migration rates, greater mixing allows the initial lakes to share alleles instead of developing their own genetic basis for adaptation.

**Figure 6:**
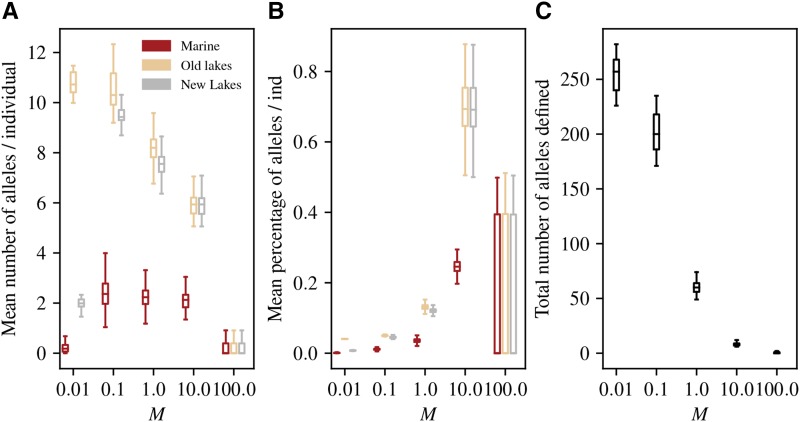
Amount of standing freshwater variation by habitat, across migration rates. Each plot counts “pre-existing freshwater adapted alleles”, that are common in the original lakes but rare in the ocean (see text for definition). (A) Mean number of these alleles per individual. (B) Mean percentage of these alleles per individual. (C) Total number of these alleles (so, B=A/C). The number of alleles meeting these conditions changes over the course of the simulation, and each plot shows distributions of these values throughout each simulations. The horizontal axis shows *M*, the mean number of migrants per lake per generation.

To investigate in more depth how locally adaptive alleles found in the original lakes are shared among newly derived lakes, as well as how they spread to the new lakes, we defined and tracked the distribution of “pre-existing freshwater adapted alleles” at the beginning of each generation. To be considered in this category, an allele must participate in the genetic basis of local adaptation for at least one of the original lakes. Concretely, these are any non-neutral mutations whose frequency is above 50% in at least one original lake and below 50% in the marine habitat. (So, “captured” alleles as defined above are pre-existing, but not all pre-existing alleles are captured.) Figure 6A shows the distribution of the number of these alleles across generations. At M=0.01, each lake has a private set of about 10 mutations nearly fixed in that lake but not elsewhere: new lakes independently acquire new adaptive alleles rather than pre-existing ones. At M=0.1, we again observe the original lakes adapting nearly independently from each other, but now the new lakes adapt using pre-existing alleles present in the original set of lakes. Concurrently, the average marine individual carries ≈2 pre-existing freshwater adapted alleles, standing variation which was nearly absent at M=0.01. As migration rate increases past this, the total number of pre-existing freshwater adapted alleles declines (Figure 6C). Interestingly, the frequency of these alleles in the ocean stays relatively constant across the reasonable rates of gene flow. We explored the possibility of dominance being a good predictor of how long an adaptive allele can be maintained in the marine environment, but found no effect strong enough to be seen with our relatively small sample size of adaptive alleles.

Figure 6B shows the distribution, through time, of the mean *percentage* of currently-defined freshwater adapted alleles that each genome in each of the populations carries. If all individuals across lakes carried the same set of alleles determining their trait value, this would be 100%. At the lowest migration rate (M=0.01), each genome in the original lakes have almost exactly $1/25^{th}$ of the total number of pre-existing freshwater adapted alleles – this is because each one of the 25 lakes has adapted with a unique set of alleles. Since these are *pre-existing* alleles, the value is zero for introduced lakes. Figure 6A shows us that at 0.1 migrants per lake per generation and above, the average individual across the new lakes has nearly the same amount of pre-existing freshwater adapted alleles as individuals across the old lakes. As expected, the genetic basis of the freshwater phenotype seems to simplify as migration increases – higher rates of migration allow adaptive alleles of larger effect to travel more efficiently through the population, even though they are deleterious in the ocean.

**Figure 7:**
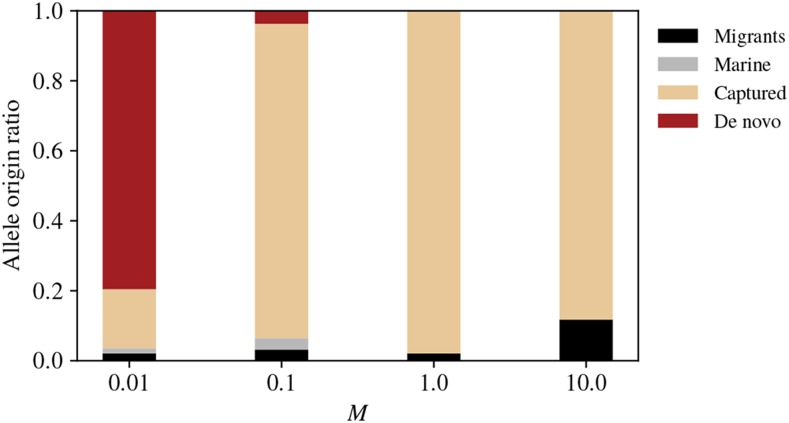
(Origin of adaptive alleles:) Each bar plot shows the origins of all trait-affecting alleles above frequency 50% in at least one new lake, classified as (red) new mutations, (black) post-colonization migrants, (tan) “captured” from pre-existing lakes, or (grey) standing marine variation. See Methods for precise definitions of these categories.

The numbers in Figure 6 strongly suggest that the dramatic increase in speed of local adaptation we observed above occurs because higher gene flow allows sharing of freshwater alleles among populations. We confirmed this finding by using recorded tree sequences to identify the origin of each trait-affecting allele in each individual in the new lakes, as defined in the Methods. Figure 7 shows that at the lowest rate of gene flow the majority of adaptive alleles are derived from “*de novo*” mutation. As gene flow increases, a larger fraction of adaptive alleles derive from pre-existing variation in the marine population at the time of introduction. In other words, greater mixing at higher migration rates allows lakes to share alleles instead of developing their own genetic basis of adaptation.

While increased migration allows sharing of adaptive alleles among lakes, at M=10.0 migrants per lake per generation the constant influx of alleles between the habitats creates substantial migration load. This level of gene flow only replaces 5% of each population each generation with migrants from the other habitat, but is sufficient to shift the mean trait values to nearly half their optimal values, as seen in Figure 2.

We find that, unsurprisingly, sharing of alleles across lakes leads to a very simple genetic basis – only tens of loci, at M≥1. This suggests that all else being equal, systems with higher migration should have fewer loci responsible for local adaptation. However, “all else is equal” is rarely plausible: the actual number of locally adaptive alleles in a particular system depends on many other factors, including the strength of selection, degree of pleiotropy, and the genetic basis of the trait. A different genetic basis could well lead to a much larger number of alleles, even at high migration rates: but, could these be reliably identified? Next, we look at how well adaptive alleles are identified with genome scans.

### The efficacy of genomic scans of selection depends on gene flow

Here, we take a closer look at the genomic architecture of local adaptation between the two habitats. Can measures of local differentiation such as $F_{ST}$ be used to identify the causal loci? This question has been extensively studied, both with simulation (*e.g.*, Southcott and Kronforst 2017; Lotterhos and Whitlock 2015) and in the wild (reviewed in Harrison and Larson 2016; Hoban *et al.* 2016); our simulations provide a detailed look where the truth is known. Figure 4 shows plots along the genome of average per-locus $F_{ST}$ values in 500bp windows between the marine habitat and all freshwater habitats pooled together. These $F_{ST}$ values are calculated using all polymorphic loci, including both those that affect phenotype as well as neutral variants. Higher rates of dispersal showed more distinct $F_{ST}$ peaks over polymorphic loci, while “background” levels of $F_{ST}$ increase as gene flow decreases, swamping out this signal until the regions under selection are indistinguishable. This is likely the contribution of two separate forces of natural selection: first, stronger genetic drift with less migration leads to higher background $F_{ST}$, and second, greater sharing of adaptive alleles providing a shared signal across populations.

At first glance, this suggests that genome scans for local adaptation based purely on measures of differentiation will only be successful given enough migration between habitats. But how many of these peaks are actually underlying trait differences that form the basis for local adaptation? To quantify this, Figure S1 shows the power and true positive rates that would be obtained by an $F_{ST}$ cutoff that declared everything above a certain value to be a causal locus.

What we observe most plainly in this graph is that M=1.0 and M=10.0 migrants per lake per generation are the only rates of dispersal at which a large percentage of peaks (above $F_{ST}=0.05$) actually lie on top of regions which impact trait differences. Unfortunately, the low statistical power for all rates of *M* would seem to suggest that many regions which impact trait differences do not appear as $F_{ST}$ peaks. In other words, with high rates of dispersal between population, $F_{ST}$ peaks may reliably identify causal loci, but not all causal loci will appear as $F_{ST}$ peaks. It’s important to note that $F_{ST}$ in Figures 4 and S1 was calculated after pooling together all lakes and all marine individuals. For comparison, Figures S2 and S4 show $F_{ST}$ calculated with individuals only in the first fifth of the range (5 lakes and corresponding marine habitat). These show a substantially lower true positive rate, as population-specific noise swamps any signal between the lakes.

As has been suggested elsewhere, introgression aids identification of locally adaptive loci from scans for differentiation. This is generally because shared adaptive alleles provide something akin to independent replicates, allowing signal to emerge above the noise created by genetic drift. However, the required sample sizes and conditions on levels of admixture may be restrictive in practice. More generally, the difficulty of correctly identifying causal loci in even our most ideal situation calls for caution (and proof-of-concept simulations) in designing and carrying out scans for alleles underlying locally adaptive, polygenic traits.

### Alleles for freshwater adaptation are mostly present in the initial generation

We have thus far found that the speed of adaptation depends strongly on the degree to which alleles can be shared between populations. However, the *origin* of the alleles underlying the phenotype is still unknown. In our simulations migration from marine individuals into the new lakes continues throughout introduction and adaptation: but is consistent influx of standing genetic variation a necessity for rapid adaptation of the population? To answer this, we traced the genealogy of *all* adaptive alleles in *all* individuals from the introduced lakes, after local adaptation. Surprisingly, for all cases with the exception of M=0.01, we found that the majority of adaptive allele origins traced back to the original generation of inhabitants in the lake (Figure 7). In our simulations, any reasonable subset of the ancestral population has the potential for rapid adaptation without the need post-colonization hybridization events.

If effectively capturing standing genetic variants is the key to rapid adaptation, as we have presented thus far, why is there a large difference in speed of adaptation between dispersal rates of M=0.1 and M=1.0 seen in Figure 5? This is surprising because we see a similar quantity of allele sharing at M=1.0, but much more rapid adaptation (≈60 *vs.* 2000 generations).

A possible explanation is that at lower rates of gene flow, freshwater alleles present as standing variation in the marine habitat are more tightly linked to marine alleles. This implies that adaptation in the new lakes must wait for recombination to separate freshwater and marine adapted alleles. A freshwater and a marine allele (of roughly equal and opposite effects) in close proximity could form an essentially neutral locus, which would persist in the marine population much longer than a freshwater allele not “masked” in this way. This suggests that a reasonable portion of freshwater alleles in the marine population would be linked to marine alleles. This genetic variation would be more difficult to access, however, so if there is sufficient additive genetic variation available to a new population in unlinked alleles, this will be used first. However, if the genetic variation initially available is not sufficient for a new population to adapt, then adaptation might need to wait for recombination to separate these haplotypes. Similar effects have been noted before in simulation (*e.g.*,Yeaman and Whitlock 2011).

In other words, perhaps higher dispersal from freshwater to the ocean maintains relatively intact freshwater haplotypes that can be more easily rebuilt in the marine environments. Recall that trait-affecting mutations only occur in relatively small regions of $10^{5}$ loci in which recombination occurs only once in every one thousand meioses. This implies that even if there is a sufficient amount of variation in the initial population of a lake to shift the trait from the marine optimum (+10) to the freshwater optimum (−10), rebuilding the most beneficial haplotype may prove to be a non-trivial task for selection. For example, suppose there are 10 variants segregating at low frequency with effect size −1 each, but each is paired with a compensatory allele with effect size +1. Each local haplotype is therefore neutral. This might also explain why at M=0.1, marine individuals still hold on average several alleles that shift the trait in the freshwater direction (Figure 6A).

To quantify the genetic variation available *without* recombination within the ten genomic regions, we first found, within each population, the haplotype with the largest net negative effect at each of the ten genomic regions. Summing these ten numbers, we get the maximum amount that selection could move the population in the freshwater direction without recombining within these regions. The mean of this value across the 25 lake populations is shown in Table 1 – typical populations at M=0.1 migrants per lake per generation could shift to a phenotype of -12.02 (and so have sufficient variation to adapt without recombination), but the “best” haplotypes in the populations at M=1.0 have effect sizes nearly twice as big at a mean total of -21.02. The amount of variation available at M=10.0 is lower (only -7.02), presumably because of migration load, while at M=0.01 almost no alleles with negative effect are present. Note that some of these haplotypes will likely be lost to drift – indeed, if they did all fix, then populations at M=0.1 would adapt much more quickly. However, this calculation supports our explanation above: it appears that adaptation at M=0.1 is slower because it must wait for intralocus recombination to free up genetic variation present but masked in the marine population.

**Table 1:** Haplotypic variation present in the new lakes at time of colonization, across rates of gene flow, “Best” quantified as the most negative trait value achievable with intact haplotypes, averaged across populations (see text for details)

	M = 0.01	M = 0.1	M = 1	M = 10
best	2.77	−12.02	−21.02	−7.02

### Simulation results align with theoretical expectations

How do our results compare to what is expected from population genetics theory? Our simulations included many loci under selection and in linkage to many other selected alleles, which makes precise calculation impossible. Nonetheless, rough calculations based on simple population genetics theory – with the benefit of hindsight – turn out to describe qualitatively most of the aspects of adaptation we observed above. Because we model stabilizing selection on an additive trait controlled by a moderately large number of loci within each population, more precise expectations might be obtained through quantitative genetics (Svardal *et al.* 2014) or even Fisher’s geometric model (Barton 2001; Chevin *et al.* 2014), but doing so is beyond the scope of this paper. The arguments we use here are standard in population genetics (see *e.g.*,Gillespie 1998).

One of the most useful things that theory tells us is about the fate of a new allele in a lake, that has appeared by either migration or mutation. If the allele has fitness advantage *s* – *i.e.*, when it is rare but present in *n* copies, the expected number of copies in the next generation is (1+s)n – then the probability that it escapes demographic stochasticity to become common in the population is approximately 2s (Lambert 2006; Haldane 1927), assuming Poisson reproduction, as we roughly have here. Since we are studying a quantitative trait, the fitness effect of each allele depends on the population context: if the individuals in the population all have trait values *z* units above the optimum, and the allele has effect size −u in heterozygotes, then the fitness advantage of the allele is the ratio of fitnesses with and without the allele. As we calculate fitness here, this is $s\left(u\right)=\text{exp}(-\beta {\left(z-u\right)}^{2})/\text{exp}\left(-\beta z^{2}\right)\approx 2\beta zu$, where β=1/450. This tells us two things: (1) the rate of adaptation decreases as the population approaches the optimum, and (2) larger mutations (in the right direction) are more likely to fix.

#### New mutations:

The total rate of appearance of new mutations per lake is $\mu_{L}=0.04$ per generation, and these are divided evenly in six categories: additive, dominant, and recessive, in either direction. Therefore, a new additive or dominant effect mutation appears once every 75 generations, on average. The effect size of each new mutation is randomly drawn from an Exponential distribution with mean 1/2, and so averaging the probability of establishment over this distribution, we get that the probability that a dominant mutation manages to establish in a population differing from the optimum by *z* is roughly ${\int^{\text{​}}}_{0}^{\infty }4\beta zu\text{exp}\left(-2u\right)du=\beta z$. Multiplying the rate of appearance of these mutations with the probability they establish predicts that the rate of establishment of dominant mutations is βz/75, *i.e.*, about one such mutation every 33750/z generations. During the initial phase of adaptation, the populations begin at around distance z=10 from the optimum, and *z* decreases as adaptation progresses. Mutations that successfully established are more likely to be strong: the distribution of the effect sizes of these successfully established mutations has density proportional to u exp(−2u), which is a Gamma distribution with mean 1 and shape parameter 2. There are also additive alleles: these have half the effect in heterozygotes, and so roughly half the probability of establishment. Combining these facts, we expect adaptive alleles to appear through mutation within lakes at first on a time scale of 3,000 generations, with the time between local fixation of new alleles increasing as adaptation progresses, and each to move the trait by a distance of order 1. This agrees roughly with what we see in Figures 3 and S3.

#### Standing variation:

How are freshwater alleles maintained in the ocean, where they are deleterious? Assuming that fish in the marine environment are close to their phenotypic optimum, an allele that when heterozygous moves the trait *u* units in the freshwater direction, has fitness roughly $\text{exp}\left(-\beta u^{2}\right)\approx 1-\beta u^{2}$, *i.e.*, a fitness differential of $s=\beta u^{2}$. The product of population size and fitness differential in the marine environment for a mutation with u=1 is therefore 2Ns≈22, implying that these alleles are strongly selected against but might drift to moderate frequency if recessive. The average frequency of such an allele in the marine environment at migration-selection equilibrium is equal to the proportion of individuals in the ocean replaced by migrants per generation divided by the selective disadvantage, *i.e.*, around $m/\beta u^{2}$. Each new lake is likely to contain a few copies of alleles at frequency above 1/400 (since each new lake is initialized with 400 randomly selected genomes). For a typical allele with effect size u=1, the equilibrium migration-selection frequency is greater than this threshold if m/β>1/400. Since 1/β=450, if $m\geq 5\times 10^{-6}$ this suggests that there is a good chance that any particular lake-adapted allele that is present in all pre-existing lakes will appear at least once in the fish that colonize a new lake. However, an allele with effect size u=1 only has probability of around 1/20 of establishing locally, and so must be present in about 20 copies to ensure establishment. Putting these calculations together, we expect migration-selection balance to maintain sufficient genetic variation for new lakes to adapt if $m\geq 10^{-4}$, which corresponds to M≥0.4. This is in good agreement with our observations. However, this calculation treats each allele independently; in practice we found that standing freshwater variation in the ocean might be masked by linkage to compensatory marine variants.

#### Migration:

The key quantity regulating the amount of standing variation in the ocean is the *downstream* migration rate, from lakes to the ocean. How important is the upstream migration rate? If sufficient genetic variation is not present in a new lake initially, it must appear either by new mutation or by migration. Since a proportion *m* of each lake is composed of migrants each generation, it takes 1/m generations until the genetic variation introduced by migrants equals the amount initially present at colonization. This implies a dichotomy: either (a) migration is high, and adaptation is possible using variants present at colonization or arriving shortly thereafter, or (b) migration is low, so adaptation takes many multiples of 1/m generations. Since in our model lower migration also reduces the amount of variation available in the ocean, we expect very little contribution of subsequent migration across any value of *m*, as seen in Figure 7.

We expect at least two other aspects of colonization and migration to be relevant. First, the size of the initial population of marine individuals colonizing each new lake affects how much of the marine genetic diversity is present. In our simulations, new lakes began with a full set of individuals, as may happen in uplifted tidal habitats. If instead each new lake began with only a few individuals (inducing an initial bottleneck), subsequent migration of alleles would likely become more relevant. If in addition, there was no subsequent “upstream” migration, adaptation might need to rely on new mutations, as at our lowest migration rates. However, exactly where these boundaries lie will require additional theoretical or simulation work.

#### Population size:

In our simulations, the marine and freshwater populations are of equal size. More generally, we expect the downstream migration rate that is required to maintain standing variation there to scale with the size of the marine population, since the same number of migrants make up a smaller proportion of a larger population. So, if the marine population was well-mixed and tenfold larger than all freshwater populations combined, the number of migrants per lake per generation would need to be ten times larger than in our simulations to maintain sufficient standing genetic variation. If the marine population was a thousandfold larger, adaptation from standing genetic variation that is deleterious in the ocean might be impossible. The fact that repeated adaptation from standing genetic variation has been shown to occur in real world threespine stickleback populations argues that the population sizes of marine and combined freshwater stickleback are not that different. While this might at first seem implausible given the ocean is large and lakes are small, there are hundreds of thousands to millions of lakes inhabited by threespine stickleback in coastal regions of the Northern Hemisphere, and the limiting habitat in the ocean constraining marine stickleback population sizes is likely to be coastal nesting areas. Together, these facts make the equivalency of marine and freshwater stickleback population sizes much more plausible.

## Conclusion

In this paper, we have analyzed a relatively realistic simulation study of a coastal meta-population to understand how repeated local adaptation operates on complex standing variation across a wide range of gene flow rates. We have shown that rapid and parallel adaptation similar to that documented in natural stickleback populations occurs over a realistic range of parameter values. These simulations therefore provide an in-depth look at (and quantitative proof-of-concept of) the “transporter” hypothesis suggested by Schluter and Conte (2009). Selection is able to rebuild the freshwater haplotype at a rapid pace (in tens of generations) from variation present at migration-selection balance in marine populations A finding of practical consequence is that the efficacy of $F_{ST}$-based genomic scans varies significantly with migration rate. Perhaps surprisingly, we found that the majority of adaptive variant can be traced back to the original generation of new habitats, with little role for post-colonization migrant alleles. In other words, a randomly chosen subset of marine stickleback carry the capacity to rapidly adapt in freshwater habitats without *any* continued connection to the rest of of the species. Thus, the large marine population was able to harbor and distribute alleles that were deleterious in the ocean habitat, but adaptive in freshwater habitats, to surrounding freshwater populations with only a few migrants per population, per generation. A few good stickleback suffice.

## References

[bib1] Barrett R. D. H., and Schluter D., 2008 Adaptation from standing genetic variation. Trends Ecol. Evol. 23: 38–44. 10.1016/j.tree.2007.09.008 18006185

[bib2] Barton N., and Bengtsson B. O., 1986 The barrier to genetic exchange between hybridising populations. Heredity 57: 357–376. 10.1038/hdy.1986.135 3804765

[bib3] Barton N. H., 2001 The role of hybridization in evolution. Mol. Ecol. 10: 551–568. 10.1046/j.1365-294x.2001.01216.x 11298968

[bib4] Bassham S., Catchen J., Lescak E., von Hippel F. A., and Cresko W. A., 2018 Repeated selection of alternatively adapted haplotypes creates sweeping genomic remodeling in stickleback. Genetics 209: 921–939. 29794240 10.1534/genetics.117.300610 PMC6028257

[bib5] Bell M., and Foster S., 1994 The Evolutionary Biology of the Three Spine Sticklebacks. pp. 1–27. Edited by Susan Adlai Foster. Oxford: Oxford University Press, 1994

[bib6] Charlesworth B., 1997 A big book and a small book on selection. Evolution 51: 1337–1338. 10.1111/j.1558-5646.1997.tb03983.x 28565501

[bib7] Charlesworth B., Charlesworth D., and Barton N. H., 2003 The effects of genetic and geographic structure on neutral variation. Annu. Rev. Ecol. Evol. Syst. 34: 99–125. 10.1146/annurev.ecolsys.34.011802.132359

[bib8] Chevin L.-M., Decorzent G., and Lenormand T., 2014 Niche dimensionality and the genetics of ecological speciation. Evolution 68: 1244–1256. 10.1111/evo.12346 24410181

[bib9] Colosimo P. F., Hosemann K. E., Balabhadra S., Villarreal G., Dickson M., 2005 Widespread parallel evolution in sticklebacks by repeated fixation of ectodysplasin alleles. Science 307: 1928–1933. 10.1126/science.1107239 15790847

[bib10] Colosimo P. F., Peichel C. L., Nereng K., Blackman B. K., Shapiro M. D., 2004 The genetic architecture of parallel armor plate reduction in threespine sticklebacks. PLoS Biol. 2: E109 10.1371/journal.pbio.0020109 15069472 PMC385219

[bib11] Cresko W. A., Amores A., Wilson C., Murphy J., Currey M., 2004 Parallel genetic basis for repeated evolution of armor loss in Alaskan threespine stickleback populations. Proc. Natl. Acad. Sci. USA 101: 6050–6055. 10.1073/pnas.0308479101 15069186 PMC395921

[bib12] Cresko W. A., McGuigan K. L., Phillips P. C., and Postlethwait J. H., 2007 Studies of threespine stickleback developmental evolution: progress and promise. Genetica 129: 105–126. 10.1007/s10709-006-0036-z 16897450

[bib13] DeFaveri J., Jonsson P. R., and Merilä J., 2013 Heterogeneous genomic differentiation in marine threespine sticklebacks: Adaptation along an environmental gradient. Evolution 67: 2530–2546. 10.1111/evo.12097 24033165

[bib14] DeFaveri J., Shikano T., Shimada Y., Goto A., and Merilä J., 2011 Global analysis of genes involved in freshwater adaptation in threespine sticklebacks (gasterosteus aculeatus). Evolution 65: 1800–1807. 10.1111/j.1558-5646.2011.01247.x 21644964

[bib15] Endler J., 1977 Geographic variation, speciation and clines. Monogr. Popul. Biol. 10: 1–246. 409931

[bib16] Feulner P. G. D., Chain F. J. J., Panchal M., Huang Y., Eizaguirre C., 2015 Genomics of Divergence along a Continuum of Parapatric Population Differentiation. PLoS Genet. 11: e1004966 10.1371/journal.pgen.1004966 25679225 PMC4334544

[bib17] Flaxman S. M., 2013 Surfing downhill: when should population range expansion be characterized by reductions in fitness? Mol. Ecol. 22: 5963–5965. 10.1111/mec.12564 24138111

[bib18] Gillespie J. H., 1998 Population Genetics, The Johns Hopkins University Press, Baltimore, MD.

[bib19] Haldane J. B. S., 1927 A mathematical theory of natural and artificial selection, part V: Selection and mutation. Math. Proc. Camb. Philos. Soc. 23: 838–844. 10.1017/S0305004100015644

[bib20] Haller B. C., Galloway J., Kelleher J., Messer P. W., and Ralph P. L., 2019 Tree-sequence recording in SLiM opens new horizons for forward-time simulation of whole genomes. Mol. Ecol. Resour. 19: 552–566. 10.1111/1755-0998.12968 PMC6393187 30565882

[bib21] Haller B. C., and Messer P. W., 2017 SLiM 2: Flexible, interactive forward genetic simulations. Mol. Biol. Evol. 34: 230–240. 10.1093/molbev/msw211 27702775

[bib22] Haller B. C., and Messer P. W., 2019 SLiM 3: Forward genetic simulations beyond the Wright-Fisher model. Mol. Biol. Evol. 36: 632–637. 10.1093/molbev/msy228 30517680 PMC6389312

[bib23] Hanson D., Barrett R. D., and Hendry A. P., 2016 Testing for parallel allochronic isolation in lake-stream stickleback. J. Evol. Biol. 29: 47–57. 10.1111/jeb.12761 26408356

[bib24] Hanson D., Hu J., Hendry A. P., and Barrett R. D. H., 2017 Heritable gene expression differences between lake and stream stickleback include both parallel and antiparallel components. Heredity 119: 339–348. 10.1038/hdy.2017.50 28832577 PMC5637370

[bib25] Harrison R. G., and Larson E. L., 2016 Heterogeneous genome divergence, differential introgression, and the origin and structure of hybrid zones. Mol. Ecol. 25: 2454–2466. 10.1111/mec.13582 26857437 PMC4899261

[bib26] Hermisson J., and Pennings P. S., 2005 Soft sweeps. Genetics 169: 2335–2352. 10.1534/genetics.104.036947 15716498 PMC1449620

[bib27] Hirase S., Ozaki H., and Iwasaki W., 2014 Parallel selection on gene copy number variations through evolution of three-spined stickleback genomes. BMC Genomics 15: 735 10.1186/1471-2164-15-735 25168270 PMC4159527

[bib28] Hoban S., Kelley J. L., Lotterhos K. E., Antolin M. F., Bradburd G., 2016 Finding the genomic basis of local adaptation: Pitfalls, practical solutions, and future directions. Am. Nat. 188: 379–397. 10.1086/688018 27622873 PMC5457800

[bib29] Hohenlohe P. A., Bassham S., Etter P. D., Stiffler N., Johnson E. A., 2010 Population genomics of parallel adaptation in threespine stickleback using sequenced RAD tags. PLoS Genet. 6: e1000862 10.1371/journal.pgen.1000862 20195501 PMC2829049

[bib30] Hunt G., Bell M. A., and Travis M. P., 2008 Evolution toward a new adaptive optimum: Phenotypic evolution in a fossil stickleback lineage. Evolution 62: 700–710. 10.1111/j.1558-5646.2007.00310.x 18081713

[bib31] Jones F. C., Grabherr M. G., Chan Y. F., Russell P., Mauceli E., 2012 The genomic basis of adaptive evolution in threespine sticklebacks. Nature 484: 55–61. 10.1038/nature10944 22481358 PMC3322419

[bib32] Kelleher J., Etheridge A. M., and McVean G., 2016 Efficient coalescent simulation and genealogical analysis for large sample sizes. PLOS Comput. Biol. 12: e1004842 10.1371/journal.pcbi.1004842 27145223 PMC4856371

[bib33] Kelleher J., Thornton K. R., Ashander J., and Ralph P. L., 2018 Efficient pedigree recording for fast population genetics simulation. PLOS Comput. Biol. 14: e1006581 10.1371/journal.pcbi.1006581 30383757 PMC6233923

[bib34] Lai Y.-T., Yeung C. K. L., Omland K. E., Pang E.-L., Hao Y., 2019 Standing genetic variation as the predominant source for adaptation of a songbird. Proc. Natl. Acad. Sci. USA 116: 2152–2157. 10.1073/pnas.1813597116 30659151 PMC6369788

[bib35] Lambert A., 2006 Probability of fixation under weak selection: a branching process unifying approach. Theor. Popul. Biol. 69: 419–441. 10.1016/j.tpb.2006.01.002 16504230

[bib36] Lescak E. A., Bassham S. L., Catchen J., Gelmond O., Sherbick M. L., 2015 Evolution of stickleback in 50 years on earthquake-uplifted islands. Proc. Natl. Acad. Sci. USA 112: E7204–E7212. 10.1073/pnas.1512020112 26668399 PMC4702987

[bib37] Lotterhos K. E., and Whitlock M. C., 2015 The relative power of genome scans to detect local adaptation depends on sampling design and statistical method. Mol. Ecol. 24: 1031–1046. 10.1111/mec.13100 25648189

[bib38] Marques D. A., Lucek K., Meier J. I., Mwaiko S., Wagner C. E., 2016 Genomics of rapid incipient speciation in sympatric threespine stickleback. PLoS Genet. 12: e1005887 10.1371/journal.pgen.1005887 26925837 PMC4771382

[bib39] Maynard Smith J. M., and Haigh J., 1974 The hitch-hiking effect of a favourable gene. Genet. Res. 23: 23–35. 10.1017/S0016672300014634 4407212

[bib41] Nelson T. C., and Cresko W. A., 2018 Ancient genomic variation underlies repeated ecological adaptation in young stickleback populations. Evolution Letters 2: 9–21. 10.1002/evl3.37 30283661 PMC6121857

[bib42] Nelson T. C., Jones M. R., Velotta J. P., Dhawanjewar A. S., and Schweizer R. M., 2019 Unveiling connections between genotype, phenotype, and fitness in natural populations. Mol. Ecol. 28: 1866–1876. 10.1111/mec.15067 30830713 PMC6525050

[bib43] Nosil P., Funk D. J., and Ortiz-Barrientos D., 2009 Divergent selection and heterogeneous genomic divergence. Mol. Ecol. 18: 375–402. 10.1111/j.1365-294X.2008.03946.x 19143936

[bib44] O’Brien E., Hunt J., and Wolf J., 2015 Quantitative genetic variation and heritability, Oxford Bibliographies in Evolutionary Biology 10.1093/OBO/9780199941728-0064

[bib45] Orr H. A., 2005 The genetic theory of adaptation: a brief history. Nat. Rev. Genet. 6: 119–127. 10.1038/nrg1523 15716908

[bib46] Petry D., 1983 The effect on neutral gene flow of selection at a linked locus. Theor. Popul. Biol. 23: 300–313. 10.1016/0040-5809(83)90020-5 6623407

[bib47] Pritchard J. K., 2010 How we are evolving. Sci. Am. 303: 40–47. 10.1038/scientificamerican1010-40 20923127

[bib48] Ralph P. L., and Coop G., 2015 The role of standing variation in geographic convergent adaptation. Am. Nat. 186: S5–S23. 10.1086/682948 26656217 PMC4827716

[bib49] Roesti M., Gavrilets S., Hendry A. P., Salzburger W., and Berner D., 2014 The genomic signature of parallel adaptation from shared genetic variation. Mol. Ecol. 23: 3944–3956. 10.1111/mec.12720 24635356 PMC4122612

[bib50] Roesti M., Hendry A. P., Salzburger W., and Berner D., 2012 Genome divergence during evolutionary diversification as revealed in replicate lake–stream stickleback population pairs. Mol. Ecol. 21: 2852–2862. 10.1111/j.1365-294X.2012.05509.x 22384978

[bib51] Samuk K., Owens G. L., Delmore K. E., Miller S. E., Rennison D. J., 2017 Gene flow and selection interact to promote adaptive divergence in regions of low recombination. Mol. Ecol. 26: 4378–4390. 10.1111/mec.14226 28667780

[bib52] Schluter D., and Conte G. L., 2009 Genetics and ecological speciation. Proc. Natl. Acad. Sci. USA 106: 9955–9962. 10.1073/pnas.0901264106 19528639 PMC2702799

[bib53] Schrider D. R., and Kern A. D., 2017 Soft sweeps are the dominant mode of adaptation in the human genome. Mol. Biol. Evol. 34: 1863–1877. 10.1093/molbev/msx154 28482049 PMC5850737

[bib54] Slatkin M., 1975 Gene flow and selection in a two-locus system. Genetics 81: 787–802. 1213276 10.1093/genetics/81.4.787 PMC1213435

[bib55] Southcott L., and Kronforst M. R., 2017 A neutral view of the evolving genomic architecture of speciation. Ecol. Evol. 7: 6358–6366. 10.1002/ece3.3190 28861239 PMC5574762

[bib56] Stuart Y. E., Veen T., Weber J. N., Hanson D., Ravinet M., 2017 Contrasting effects of environment and genetics generate a continuum of parallel evolution. Nat. Ecol. Evol. 1: 0158 10.1038/s41559-017-0158 28812631

[bib57] Svardal, H., C. Rueffler, and J. Hermisson, 2014 A general condition for adaptive genetic polymorphism in temporally and spatially heterogeneous environments. arxiv:1411.3709. 10.1016/j.tpb.2014.11.002 25446960

[bib58] Terekhanova N. V., Logacheva M. D., Penin A. A., Neretina T. V., Barmintseva A. E., 2014 Fast evolution from precast bricks: Genomics of young freshwater populations of threespine stickleback *Gasterosteus aculeatus*. PLoS Genet. 10: e1004696 10.1371/journal.pgen.1004696 25299485 PMC4191950

[bib59] Thompson J. N., 1997 Evaluating the dynamics of coevolution among geographically structured populations. Ecology 78: 1619–1623. 10.1890/0012-9658(1997)078[1619:ETDOCA]2.0.CO;2

[bib60] Yeaman S., and Whitlock M. C., 2011 The genetic architecture of adaptation under migration-selection balance. Evolution 65: 1897–1911. 10.1111/j.1558-5646.2011.01269.x 21729046

